# LL-37-Induced Autophagy Contributed to the Elimination of Live *Porphyromonas gingivalis* Internalized in Keratinocytes

**DOI:** 10.3389/fcimb.2020.561761

**Published:** 2020-10-15

**Authors:** Xue Yang, Li Niu, Yaping Pan, Xianghui Feng, Jie Liu, Yan Guo, Chunling Pan, Fengxue Geng, Xiaolin Tang

**Affiliations:** ^1^Department of Periodontology, School and Hospital of Stomatology, China Medical University, Shenyang, China; ^2^Liaoning Provincial Key Laboratory of Oral Diseases, School of Stomatology, China Medical University, Shenyang, China; ^3^Department of Periodontology, Peking University School and Hospital of Stomatology, Beijing, China; ^4^Center of Science Experiment, China Medical University, Shenyang, China; ^5^Department of Oral Biology, School of Stomatology, China Medical University, Shenyang, China

**Keywords:** *Porphyromonas gingivalis*, internalization, LL-37, autophagy, keratinocytes, transcriptome sequencing

## Abstract

*Porphyromonas gingivalis* (*P. gingivalis*), one of the most important pathogens of periodontitis, is closely associated with the aggravation and recurrence of periodontitis and systemic diseases. Antibacterial peptide LL-37, transcribed from the cathelicidin antimicrobial peptide (*CAMP*) gene, exhibits a broad spectrum of antibacterial activity and regulates the immune system. In this study, we demonstrated that LL-37 reduced the number of live *P. gingivalis* (ATCC 33277) in HaCaT cells in a dose-dependent manner via an antibiotic-protection assay. LL-37 promoted autophagy of HaCaT cells internalized with *P. gingivalis*. Inhibition of autophagy with 3-methyladenine (3-MA) weakened the inhibitory effect of LL-37 on the number of intracellular *P. gingivalis*. A cluster of orthologous groups (COGs) and a gene ontology (GO) functional analysis were used to individually assign 65 (10%) differentially expressed genes (DEGs) to an “Intracellular trafficking, secretion, and vesicular transport” cluster and 306 (47.08%) DEGs to metabolic processes including autophagy. Autophagy-related genes, a tripartite motif-containing 22 (*TRIM22*), and lysosomal-associated membrane protein 3 (*LAMP3*) were identified as potentially involved in LL-37-induced autophagy. Finally, bioinformatics software was utilized to construct and predict the protein–protein interaction (PPI) network of CAMP-TRIM22/LAMP3-Autophagy. The findings indicated that LL-37 can reduce the quantity of live *P. gingivalis* internalized in HaCaT cells by promoting autophagy in these cells. The transcriptome sequencing and analysis also revealed the potential molecular pathway of LL-37-induced autophagy.

## Introduction

*Porphyromonas gingivalis (P. gingivalis)*, a keystone periodontal pathogen, is a Gram-negative bacterium with a variety of virulence factors (Shah and Collins, 1988). It is closely associated with the aggravation and relapse of periodontitis (Socransky and Haffajee, 1992; Grossi et al., 1995; Holt and Ebersole, 2005). The epithelium of oral mucosa can be divided into keratinized and non-keratinized mucosa. Keratinized epithelium consists of keratinocytes, such as gingival epithelium. These epithelial tissues are the first defense against bacterial invasion. *P. gingivalis* can invade epithelial cells widely (Yilmaz et al., 2006). It has even been found that *P. gingivalis* can regulate the cell cycle process and the expression of inflammatory factors after it is internalized in immortalized gingival epithelial cells (Pan et al., 2014). Moreover, it has a potential impact on the malignant transformation of gingival epithelial cells (Chang et al., 2019a,b; Geng et al., 2019). In addition, *P. gingivalis* can cause a latent case of human immunodeficiency virus-1 (HIV-1) and mediate HIV-1 in Hela epithelial cells, also a kind of keratinocyte (Imai et al., 2009; Mantri et al., 2014). Therefore, it is important to kill *P. gingivalis* when it is internalized in keratinocytes. Broad-spectrum antibiotics used for this purpose have many side effects, such as dysbacteriosis and antibiotic resistance (Soares et al., 2012). As a common drug used to inhibit anaerobes, metronidazole cannot penetrate into mammalian cells to inhibit intracellular bacteria (Eick et al., 2004; Löfmark et al., 2010). As such, effective drugs that can inhibit intracellular *P. gingivalis* with minimal side effects must be identified urgently.

Autophagy is the process whereby cells phagocytize their own organelles or cytoplasm and finally degrade cargos in lysosomes (Weidberg et al., 2011). It plays a vital role in stress response, immune defense, and homeostasis, and it is an important defense against invading microorganisms (Sanjuan and Green, 2008; Lapierre et al., 2011). Studies have indicated that *P. gingivalis* can promote autophagy in THP-1 cells, suggesting that autophagy can also promote the clearance of *P. gingivalis* in phagocytes (Park et al., 2017). Lamont et al. (1995) found that *P. gingivalis* mainly existed in a free state in gingival epithelial cells. Our previous study found that *P. gingivalis* mainly existed in a free state, but it was enclosed by incomplete autophagosomes in KB cells, a type of Hela cell subline, suggesting that *P. gingivalis* may escape capture by autophagy and promote the formation of incomplete autophagosomes in epithelial cells through some mechanism (Hu et al., 2019). Furthermore, drugs that can regulate autophagy process may help eliminate intracellular *P. gingivalis*. However, the molecular mechanism of the effects of autophagy on *P. gingivalis* in epithelial cells remains unknown.

Human cationic antimicrobial peptide-18 (hCAP18) is the only antimicrobial peptide (AMP) in the cathelicidins family found in the human body that is transcribed from the human cathelicidin antimicrobial peptide (*CAMP*) gene. It is the precursor of LL-37, which mainly exists in neutrophils and monocyte macrophages (Vandamme et al., 2012). LL-37 exhibits broad-spectrum antibacterial activity against Gram-positive and Gram-negative bacteria, fungi, and envelope viruses (Larrick et al., 1995; Dorschner et al., 2001; Hase et al., 2003; Wang et al., 2008). LL-37 protein contains hydrophobic N-terminal and hydrophilic C-terminal (Burton and Steel, 2009), and it can bind and neutralize lipopolysaccharide (LPS) and destroy the cell wall of bacteria, thus demonstrating a direct antibacterial effect (Larrick et al., 1995; Turner et al., 1998). Apart from this effect, LL-37 has been shown to antagonize intracellular *Mycobacterium* by promoting autophagy in macrophages. Rekha et al. (2015) found that endogenous LC3 could be co-localized with hCAP-18/LL-37 in autophagosomes to induce autophagy and limit the growth of *Mycobacterium tuberculosis* in macrophages. Yuk et al. (2009) found that LL-37 could induce autophagy in human monocytes, promote the expression of the autophagy-related proteins Beclin-1 and LC3, and induce the colocalization of *Mycobacterium tuberculosis* with autophagosomes in cells. In addition, Wan et al. (2018) found that LL-37 could inhibit the number of *Mycobacterium tuberculosis* in macrophages by promoting autophagy. However, it has been rarely reported whether LL-37 can help eliminate bacteria in keratinocytes, such as gingival epithelial keratinocytes. In addition, it is not clear whether LL-37 participates in the elimination of intracellular *P. gingivalis* in keratinocytes.

In this study, we used the human immortalized epidermal keratinocyte HaCaT cell line as a study model. These cells share similar morphological characteristics with gingival epithelial cells and have been frequently used as study models of gingival epithelial cells (de Camargo Pereira et al., 2013; Kidwai et al., 2013; Kim et al., 2018). The purpose of this study was to investigate the effect of LL-37 on *P. gingivalis* internalized in HaCaT cells, the possible role of autophagy, and the potential molecular pathway during this process. The findings of the present study may provide new clues for the clearance of *P. gingivalis* in gingival keratinocytes.

## Materials and Methods

### Antibodies, Chemicals, and Plasmids

The primary antibodies for LC3B (14600-1) and GAPDH (10494-1) were from Proteintech (Rosemont, USA). LL-37 (ab180760) and Anti-SQSTM1/p62 (ab207305) were from Abcam (Massachusetts, USA). The DyLight 800-labeled secondary antibody (A23220) was from Abbkine (California, USA). In addition, 3-Methyladenine (3-MA) was purchased from Selleck (Texas, USA). Transfection reagents GoldenTran-D were purchased from Golden Trans Technology (Jilin, China). The transient plasmid containing *CAMP* cDNA and the empty vector were from Genepharma (Suzhou, China).

### Bacteria and Bacterial Culture

The *P. gingivalis* ATCC 33277 strain was originally obtained from the American Tissue Culture Collection (Maryland, USA) and stored at the Department of Oral Biology at China Medical University. The bacteria were maintained anaerobically at 37°C on brain-heart-infusion (BHI) Ager medium plates, supplemented with 5% sterilized and defibrinated sheep blood, 0.5% hemin, and 0.1% Vitamin K. All bacterial culture reagents were purchased from Aoboxing Bio-tech (Beijing, China). Then, *P. gingivalis* was cultured in a liquid BHI medium for 16–18 h. An optical density of 1.0 at 600 nm (NanoDrop2000, Keyu Xingye Technology Development Co., Ltd., Beijing, China) for *P. gingivalis* was determined to correlate to 10^9^ bacteria/mL.

### Cell Lines and Cell Culture

The human keratinocyte cell line HaCaT was obtained from the Cell Resource Center at the Institute of Basic Medical Sciences, CAMS/PUMC (Beijing, China). HaCaT cells were maintained in α-MEM supplemented with 10% fetal bovine serum (FBS) under the conditions of 37°C with 5% CO_2_. Upon reaching 80% confluent growth, the HaCaT cells were dissociated with 0.05% trypsin-EDTA and resuspended by gentle pipetting in fresh complete media. In addition, 0.05% trypsin-EDTA was purchased from Gibco Laboratories (NY, USA), and the other cell culture regents were purchased from HyClone Laboratories (Logan, UT, USA).

### Establishment of the *P. gingivalis* Internalized HaCaT by an Antibiotic Protection Assay

A bacterial internalization model was established by an antibiotic protection assay (Lamont et al., 1995). The HaCaT cells were infected with *P. gingivalis* with a specific multiplicity of infection (MOI) for 6 h in antibiotic-free α-MEM. Then, cells were washed three times with sterile Phosphate Buffered Saline (PBS, Hyclone, Logan, UT, USA) and were further incubated in the fresh culture medium containing 300 μg/ml of gentamicin and 200 μg/ml of metronidazole (Sigma, St. Louis, MO, USA) for an additional 1.5 h.

### Transfection Assays

For transfection, the HaCaT cells were plated on six-well, flat-bottom plates at a seeding density of 3 × 10^5^, and grown to 80% confluence. For transient overexpression of LL-37, the LL-37 plasmid was transfected into HaCaT cells for 6 h. An empty vector was used as the control. The instructions to complete cell transfection were followed. Then, we replaced the medium with fresh serum-free α-MEM. The cells transfected with plasmids were used in subsequent experiments.

### Quantitative Real-Time Polymerase Chain Reaction

Total RNA was extracted from cells using TRIzol reagent (Invitrogen Life Technologies, Gaithersburg, MD, USA) according to the manufacturer's instructions. Complementary DNA (cDNA) was then synthesized using 2 μg of the total RNA according to the instructions in the reverse transcriptase kit (Takara Bio, Inc., Dalian, China). Real-time PCR analyses were conducted on an ABI Prism 7500 Sequence Detection System (Applied Biosystems, Foster City, CA, USA) in combination with a SYBR Premix Ex TaqTM II PCR Master Mix Reagents kit (Takara Bio, Inc., Dalian, China). Amplification was performed under the following cycling conditions: preincubation of 95°C for 30 s followed by 40 cycles at 95°C for 5 s and at 60°C for 34 s. Primers were designed and synthesized by Shanghai Sango Biotech Co. Ltd. (Shanghai, China) (Table 1). The data for *P. gingivalis* 16S rRNA were analyzed according to the absolute quantification method. Furthermore, other data were analyzed according to relative quantification. The cycle threshold (Ct) of different genes was first normalized to GAPDH for the same sample, and fold changes were calculated through relative quantification (2^−ΔΔCt^) as previously reported. Each experiment was performed in triplicate.

**Table 1:** Primers used for real-time PCR.

**Primers**	**Sequences (5^**′**^-3^**′**^)**
*Porphyromonas*	Forward Primer: AGGCAGCTTGCCATACTGCG
*gingivalis* 16S rRNA	Reverse Primer: ACTGTTAGCAACTACCGATG
LL-37	Forward Primer: TCGGATGCTAACCTCTACCG
HIF1A-AS2 TRIM22 PRKCQ LAMP3 ATP6V1B1 IGBP1-AS1	Reverse Primer: GGGTACAAGATTCCGCAAAA Forward Primer: GATGGAAGCACTAGACAAAGTTCA Reverse Primer: ATCAGTGGTGGCAGTGGTAGTG Forward Primer: CGACCTAATCGGCATCTGGCCA Reverse Primer: CCTCGTTTATGCGGAATGTTTGGTG Forward Primer: TGGAAAGTGAGAGGGAAGGTTTGC Reverse Primer: GCTGAGAATGGGTGGATGGAAAGG Forward Primer: CCACACCCAACAACTCACAC Reverse Primer: CTGGAAGGGTGGTCTGGTTA Forward Primer: GCTGGACCTGAAGTCTCAGAGC Reverse Primer: CCCAGGCCTGCTGTCTATCTC Forward Primer: GGCATCAACTTCTAACTCATCTCG Reverse Primer: CTCATACCAGTCACTCACCGTCAT
GAPDH	Forward Primer: GAAGGTGAAGGTCGGAGTC
	Reverse Primer: GAAGATGGTGATGGGATTTC

### Western Blotting

The HaCaT cells were lysed with an RIPA lysis buffer supplemented with 1 mM PMSF. The protein concentration was quantified using the BCA reagent (Beyotim, P0012, Shanghai, China). Proteins were separated by SDS-polyacrylamide gel electrophoresis (SDS-PAGE; Bio-Rad, Hercules, CA, USA) and transferred onto a PVDF membrane (0.22 μm). The membranes were then incubated with primary antibodies (LC3B, p62, and LL-37) at 4°C overnight. GAPDH was used as the internal control. Membranes were incubated with the secondary antibody in the dark at 25°C for 60 min. The images were obtained by the Infrared Fluorescence Scanning Imaging System (Odyssey CLx, LI-COR, USA). The density of the protein bands was measured and analyzed using Image J 1.52v software (NIH Image, Bethesda, MD, USA).

### High-Throughput Sequencing

The mRNA sequencing and eukaryotic reference transcriptome analysis were further performed at Majorbio Technology Co., Ltd. (Shanghai, China) using the Illumina Novaseq 6000 platform (Illumina, San Diego, CA, USA).

### Analysis of Sequencing Data

The raw data obtained from the high-throughput read were filtered using SeqPrep and Sickle software to remove connector contamination and low-quality and unknown reads. RSEM, Kallisto, and Salmon software were used to quantitatively analyze the expression levels of genes and transcripts. The FPKM, TPM, and RPM methods were used to measure the level of expression. Differentially expressed genes (DEGs) were screened using DESeq2, DEGseq, and edgeR software (absolute value of fold change ≥ 2, corrected *P* < 0.05). Gene Ontology (GO) enrichment analysis of functionally significant terms in the GO database was applied using Goatools software and the Fisher exact test (corrected *P* < 0.05) to find significantly enriched GO terms in DEGs by comparing them to the genome background. For the Kyoto Encyclopedia of Genes and Genomes (KEGG) pathway enrichment analysis, we mapped all of the DEGs to terms in the KEGG database (http://www.genome.jp/kegg/), looking for significantly enriched KEGG terms. We then analyzed the terms by Fisher's exact test using R script (corrected *P* < 0.05). Similarly, the Cluster of Orthologous Group (COG) analysis was performed in the COG database (http://www.ncbi.nlm.nih.gov/COG/).

### The Protein-Protein Interaction (PPI) Network Construction

In order to further explore the interaction network of the *CAMP* with identified genes and identified genes with autophagy, the Network Data Exchange (NDEx, http://www.ndexbio.org/) was used to investigate the possible networks (Pratt et al., 2015; Pillich et al., 2017). NDEx is a searchable collection of gene expression and protein–protein interaction networks from multiple network and pathway databases, including the Biological General Repository for Interaction Datasets (BioGRID, https://thebiogrid.org/) (Oughtred et al., 2019), Human Integrated Protein-Protein Interaction Reference (HIPPIE, http://cbdm-01.zdv.unimainz.de/~mschaefer/hippie/) (Alanis-Lobato et al., 2017), and Search Tool for the Retrieval of Interacting Genes (STRING, https://string-db.org/) (Szklarczyk et al., 2017). The Cytoscape software (version 3.8.0, http://www.cytoscape.org/), which integrates the CyNDEx App, was used to construct and visualize the PPI network (Shannon et al., 2003). The interaction was considered statistically significant when the combined score was >0.4.

### Statistical Analysis

All of the data were expressed as the means ± standard deviation (S.D.). Differences among the groups were analyzed by one-way analysis of variance (ANOVA) in the SPSS 22.0 software package (SPSS Inc., Chicago, IL, USA). For *p* values, *P* < 0.05 in comparison with the control was considered to be statistically significant. The data were representative of triplicate experiments.

## Results

### LL-37 Reduced the Quantity of Live *P. gingivalis* Internalized in HaCaT Cells in a Dose-Dependent Manner

**Figure 1:**
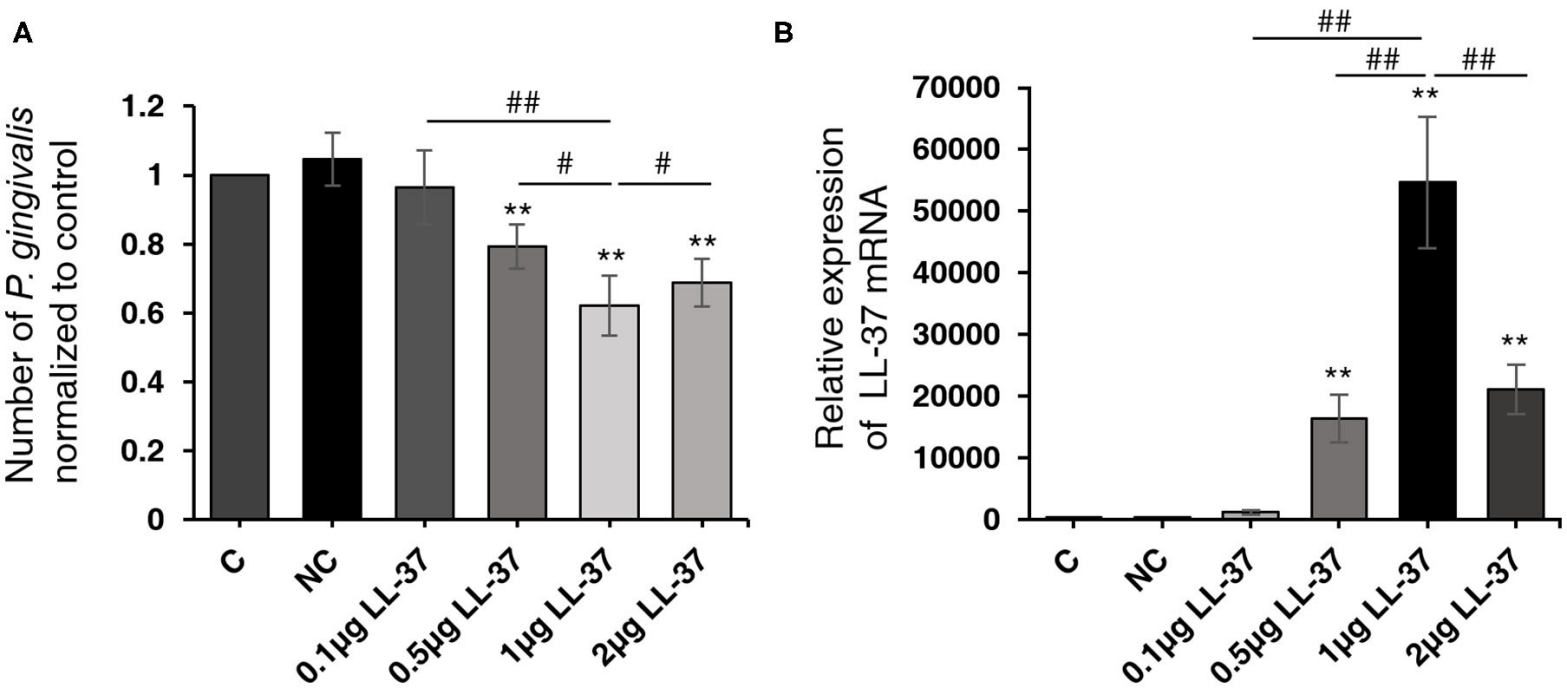
LL-37 reduced the quantity of live *Porphyromonas gingivalis* internalized in HaCaT cells in a dose-dependent manner. HaCaT cells transfected with LL-37 plasmids (0.1, 0.5, 1, or 2 μg, 6 h) or empty vectors (2 μg, 6 h) were internalized with *Porphyromonas gingivalis* (*P. gingivalis*, MOI 100, 6 h) using the antibiotic protection assay, and the total RNA was extracted after 18 h. The number of *P. gingivalis* and the expression of LL-37 mRNA were detected with qRT-PCR. **(A)** The number of live *P. gingivalis* in HaCaT cells. **(B)** The relative expression of LL-37 mRNA in HaCaT cells. The data of *P. gingivalis* 16S rRNA were analyzed according to the absolute quantification method. The data of LL-37 mRNA were analyzed according to the relatively quantification. Data presented as the mean ± standard deviation (*n* = 3) relative to control are shown in bar graphs. C: untreated control, HaCaT cells that were not transfected; NC: negative control, HaCaT cells transfected with empty vectors; 0.1, 0.5, 1, or 2 μg LL-37: HaCaT cells transfected with 0.1, 0.5, 1, or 2 μg of LL-37 plasmids. **P < 0.01, compared with the negative control. ^#^*P* < 0.05 and ^*##*^*P* < 0.01, compared with 1 μg of LL-37 plasmids transfection.

To investigate the effect of over-expressing LL-37 on the number of live *P. gingivalis* internalized in cells, HaCaT cells were transfected with a 2 μg of empty vector and 0.1, 0.5, 1, or 2 μg of LL-37 plasmids for 6 h and then internalized with *P. gingivalis* (MOI 100, 6 h) using the antibiotic protection assay. Total RNA was extracted after 18 h. The qRT-PCR results showed that compared with the control cells, the number of live *P. gingivalis* was significantly reduced (*P* < 0.01) by 0.79, 0.62, and 0.69 times in cells transfected with 0.5, 1, or 2 μg of LL-37 plasmids, respectively (Figure 1A). *LL-37* mRNA expression gradually increased significantly as transfected with 0.1, 0.5, or 1 μg of LL-37 plasmids, which was followed by a decrease and reached a peak in cells treated with 1 μg of LL-37 plasmids (Figure 1B).

### *P. gingivalis* Internalization Induced Autophagy

**Figure 2:**
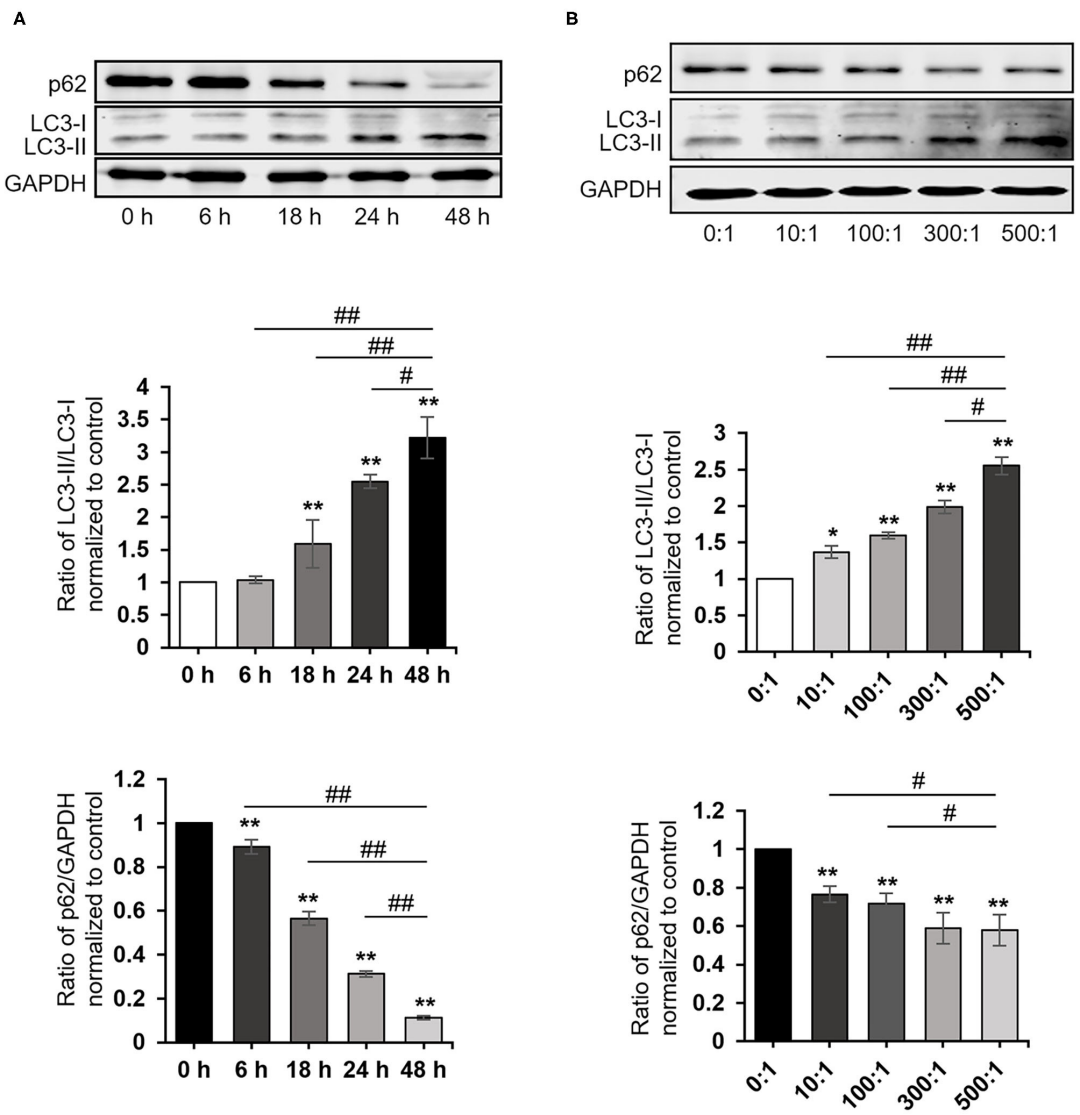
*Porphyromonas gingivalis* internalization induced autophagy. The ratio of LC3-II/LC3-I, p62, and GAPDH were detected by a Western blot analysis of cell lysates from HaCaT cells treated as follows: **(A)** Taking *P. gingivalis*-internalized HaCaT cells (MOI 100, 6 h) by the antibiotic-protection assay as the starting point, the total protein was extracted after 0, 6, 18, 24, and 48 h, respectively. **(B)** HaCaT cells were internalized with *P. gingivalis* (MOI 10–500, 6 h) by the antibiotic protection assay, and the total protein was extracted after 18 h. Protein-band density was analyzed using an NIH ImageJ software. Data presented as the mean ± standard deviation (*n* = 3) relative to control are shown in bar graphs. *P < 0.05 and **P < 0.01, compared with control; ^#^*P* < 0.05 and ^##^*P* < 0.01, compared with *P. gingivalis* infection for 48 h **(A)** or 500 MOI of *P. gingivalis* infection **(B)**.

To explore whether *P. gingivalis* induces autophagy in cells, HaCaT cells were treated as follows: (A) Taking *P. gingivalis* internalized HaCaT cells (MOI 100, 6 h) by the antibiotic-protection-assay as the starting point, the total protein was extracted after 0, 6, 18, 24, and 48 h. (B) HaCaT cells were internalized with *P. gingivalis* (MOI 10–500, 6 h) using the antibiotic protection assay, and the total protein was extracted after 18 h. Western blot assays demonstrated that *P. gingivalis* significantly increased the ratio of LC3-II/LC3-I and decreased the expression of p62 in HaCaT cells in a dose- and time-dependent manner, where *P* < 0.01 (Figures 2A,B).

### LL-37 Induced Autophagy

**Figure 3:**
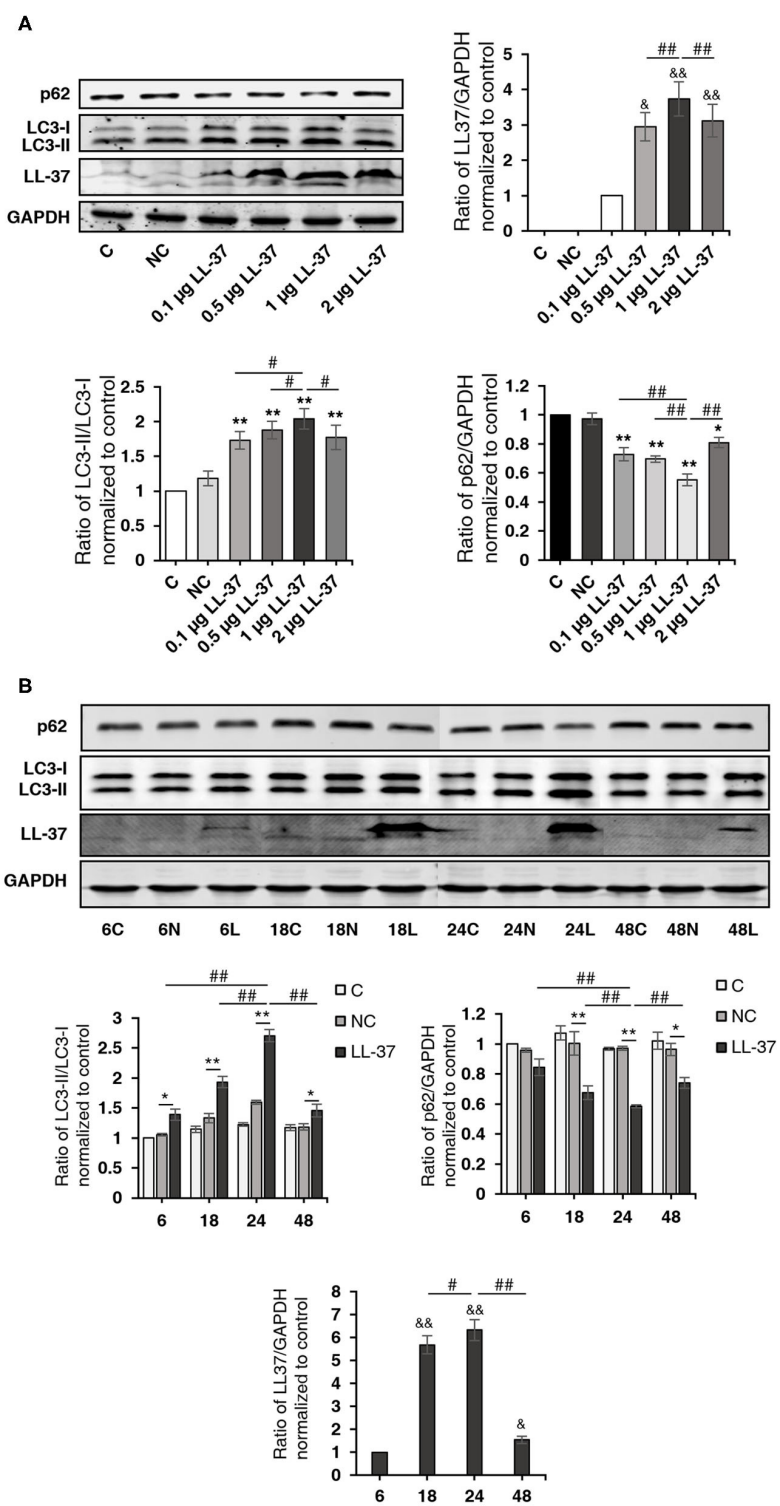
LL-37 promoted autophagy. The ratio of LC3-II/LC3-I, p62, LL-37, and GAPDH were detected by a Western blot analysis of cell lysates from HaCaT cells transfected with **(A)** 0.1, 0.5, 1, or 2 μg of LL-37 plasmids or 2 μg of empty vectors for 24 h or **(B)** LL-37 plasmids (1 μg) or empty vectors (1 μg) for 6–48 h. NC: negative control, HaCaT cells transfected with empty vectors. *P < 0.05 and **P < 0.01, compared with negative control; ^#^*P* < 0.05 and ^##^*P* < 0.01, compared with 1 μg of LL-37 plasmids transfection for 24 h; ^&^*P* < 0.05 and ^&&^*P* < 0.01, compared with 0.1 μg LL-37 plasmids transfection **(A)** or LL-37 plasmids (1 μg) transfection for 6 h **(B)**.

To determine whether LL-37 induces autophagy in cells, HaCaT cells were transfected and treated with LL-37 plasmids (0.1, 0.5, 1, or 2 μg) for 24 h and LL-37 plasmids (1 μg) for 6–48 h. Western blot assays demonstrated that LL-37 significantly increased the ratio of LC3-II/LC3-I and decreased the expression of p62 in HaCaT cells in a dose- and time-dependent manner, where *P* < 0.05 (Figure 3).

**Figure 4:**
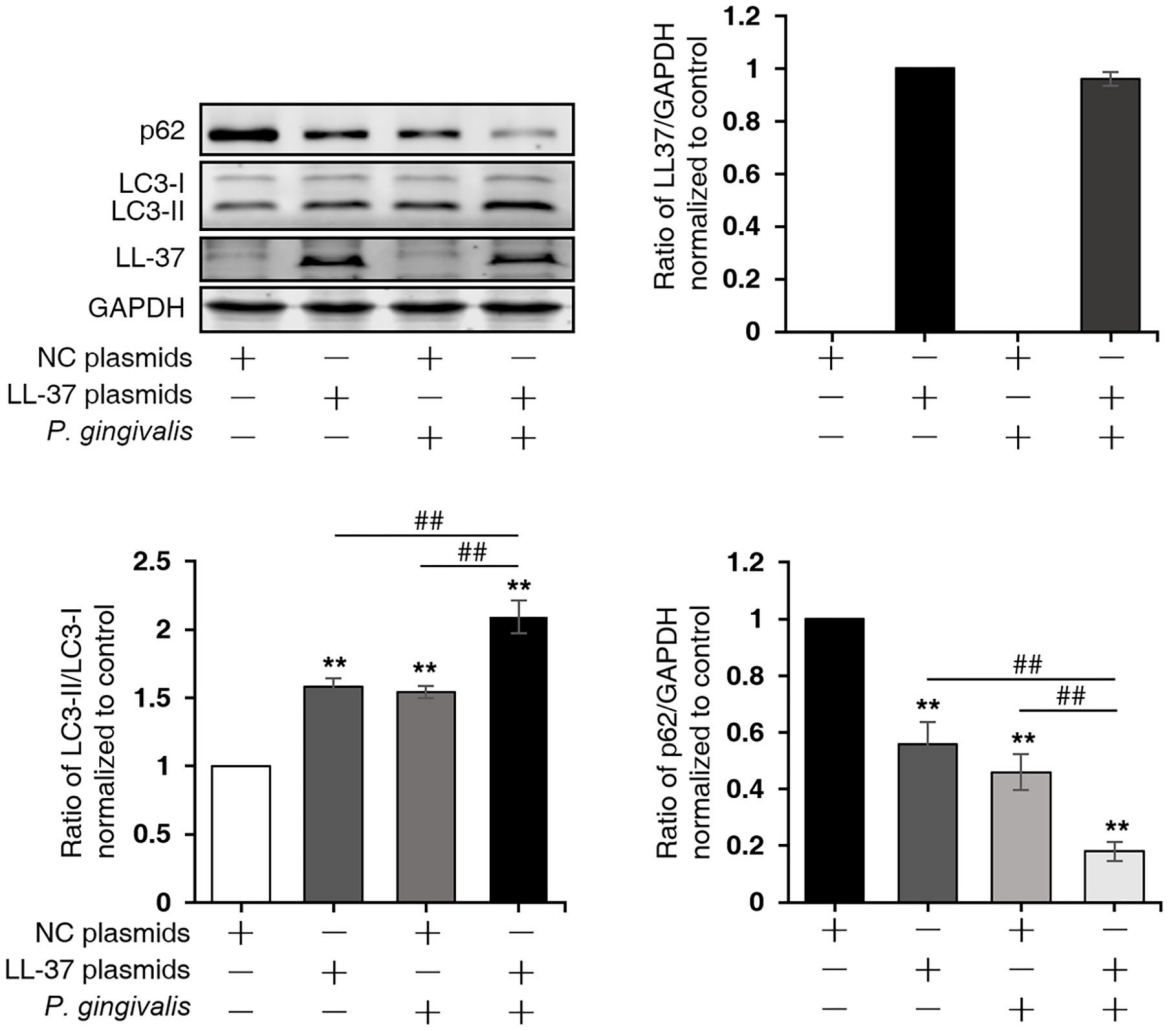
LL-37 induced autophagy in *P. gingivalis* internalized HaCaT cells. HaCaT cells were divided into four groups: negative control (NC), LL-37, NC+*P. gingivalis*, and LL-37+*P. gingivalis*. Cells were transfected with 1 μg of empty vectors (NC and NC+*P. gingivalis* groups) or 1 μg of LL-37 plasmids (LL-37 and LL-37+*P. gingivalis* groups) for 6 h. Then, cells in the NC+*P. gingivalis* and LL-37+*P. gingivalis* groups were internalized with *P. gingivalis* (MOI 100, 6 h) by the antibiotic protection assay, the total protein was extracted after 18 h. The ratio of LC3-II/LC3-I, p62, LL-37 and GAPDH were detected by Western blot assays. **P < 0.01, compared with negative control; ^##^*P* < 0.01, compared with 1 μg of LL-37 plasmids and 100 MOI of *P. gingivalis* treatment.

To investigate the effect of LL-37 on autophagy in *P. gingivalis* internalized HaCaT cells, cells were divided into four groups: NC, LL-37, NC+*P. gingivalis*, and LL-37+*P. gingivalis*. Cells were transfected with 1 μg of empty vectors (NC group and NC+*P. gingivalis* group) or 1 μg of LL-37 plasmids (LL-37 and LL-37+*P. gingivalis* groups) for 6 h. Then, cells in the NC+*P. gingivalis* and LL-37+*P. gingivalis* groups were internalized with *P. gingivalis* (MOI of 100, 6 h) using the antibiotic protection assay, and the total protein was extracted after 18 h. Western blot assays demonstrated that *P. gingivalis* and LL-37 significantly increased the ratio of LC3-II/LC3-I and decreased the expression of p62 in HaCaT cells, where *P* < 0.01 (Figure 4).

### Autophagy Inhibition Significantly Decreased the Antibacterial Effect of LL-37

**Figure 5:**
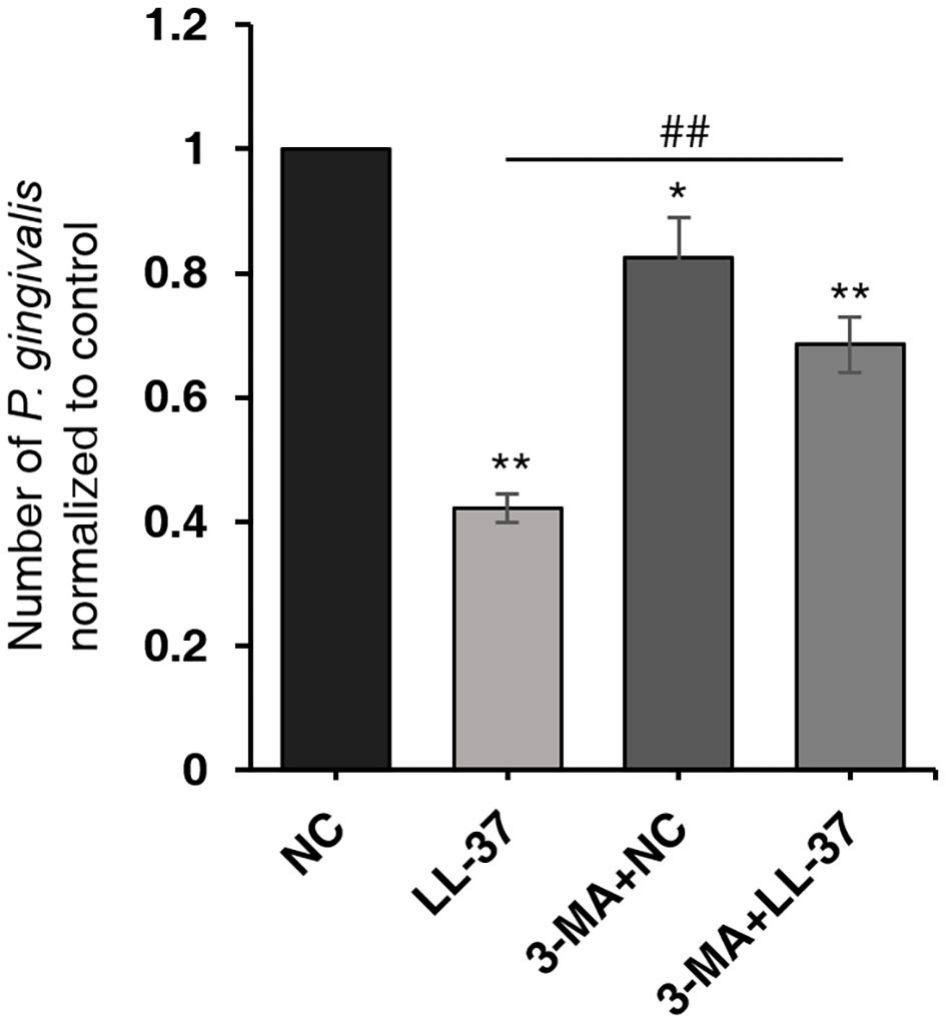
Autophagy inhibition significantly decreased the antibacterial effect of LL-37. HaCaT cells were divided into four groups: NC, LL-37, 3-MA+NC, and 3-MA+LL-37. In the 3-MA+LL-37 and 3-MA+NC groups, the cells were pretreated with 3-MA (10 mM, 3 h). Then, cells in the four groups that were transfected with LL-37 plasmids or empty vectors (1 μg, 6 h) were internalized with *P. gingivalis* (MOI 100, 6 h) using the antibiotic protection assay. The total RNA was then extracted after 18 h. The qRT-PCR method was used to detect the numbers of live *P. gingivalis* in HaCaT cells. *P < 0.05 and **P < 0.01, compared with negative control; ^##^*P* < 0.01, compared with LL-37 plasmids treatment.

To further verify the effect of autophagy on the number of *P. gingivalis* in cells, HaCaT cells were divided into four groups: NC, LL-37, 3-MA+NC, and 3-MA+LL-37. In the 3-MA+LL-37 and 3-MA+NC groups, the cells were pretreated with 3-MA (10 mM, 3 h). Then, cells in the four groups transfected with LL-37 plasmids or with empty vectors (1 μg, 6 h) were internalized with *P. gingivalis* (MOI 100, 6 h) using the antibiotic protection assay, and the total RNA was extracted after 18 h. The qRT-PCR result revealed that compared with that in the NC group, the number of *P. gingivalis* in the 3-MA+NC group was significantly decreased by 17.51% after the inhibition of autophagy, where *P* < 0.05. Moreover, the number of *P. gingivalis* in the 3-MA+LL-37 group significantly increased by 162.57% after the inhibition of autophagy compared with that in the LL-37 group, where *P* < 0.01 (Figure 5).

### DEGs Analysis

**Figure 6:**
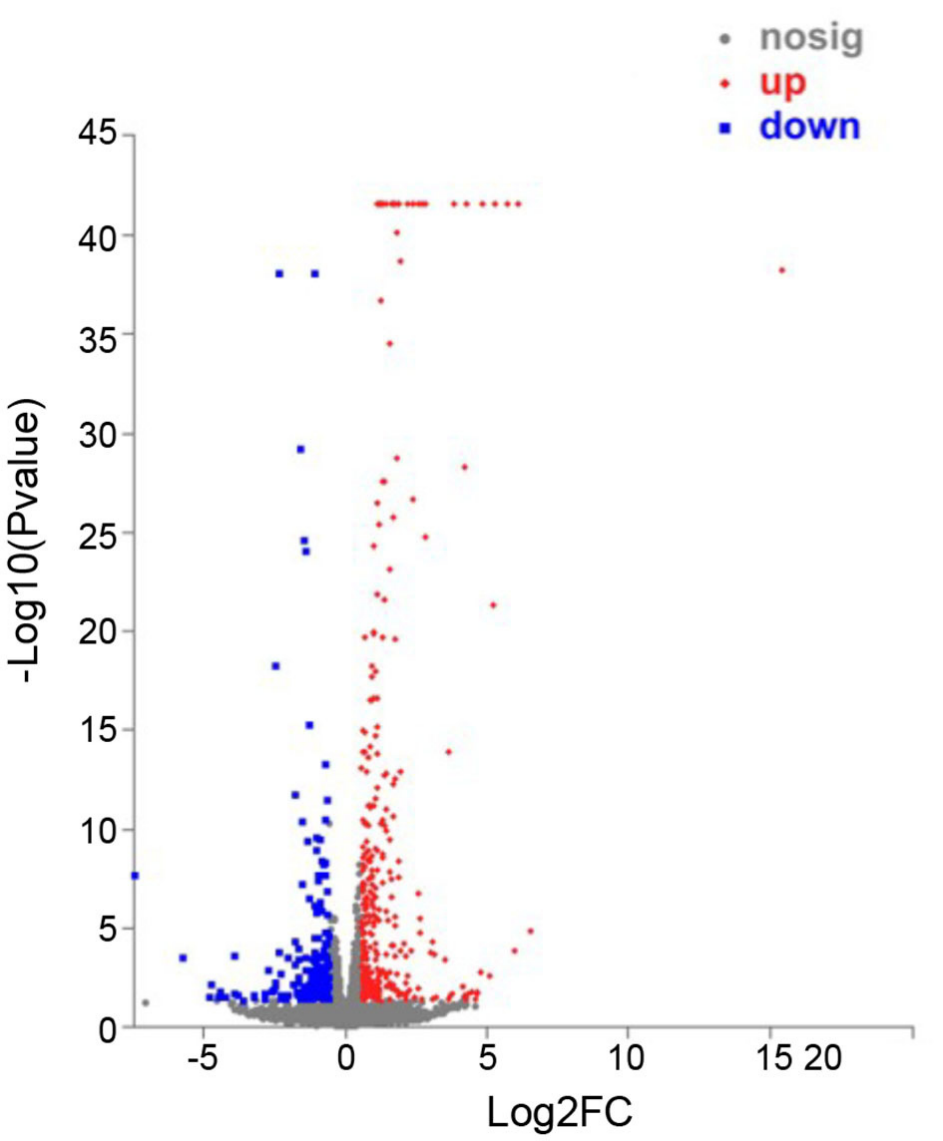
Scatter plot analysis of differentially expressed genes (DEGs) in NC-vs-LL-37. The x-axis is the fold-change value of the DEGs between the two samples. The y-axis shows that the larger the ordinate-log10 (*P*-value), the more significant the expression difference. Compared with the specific genes, the dots in the figure are significantly up-regulated as red dots, significantly down-regulated as blue dots, and non-significantly different genes are gray dots. *P* < 0.05.

**Figure 7:**
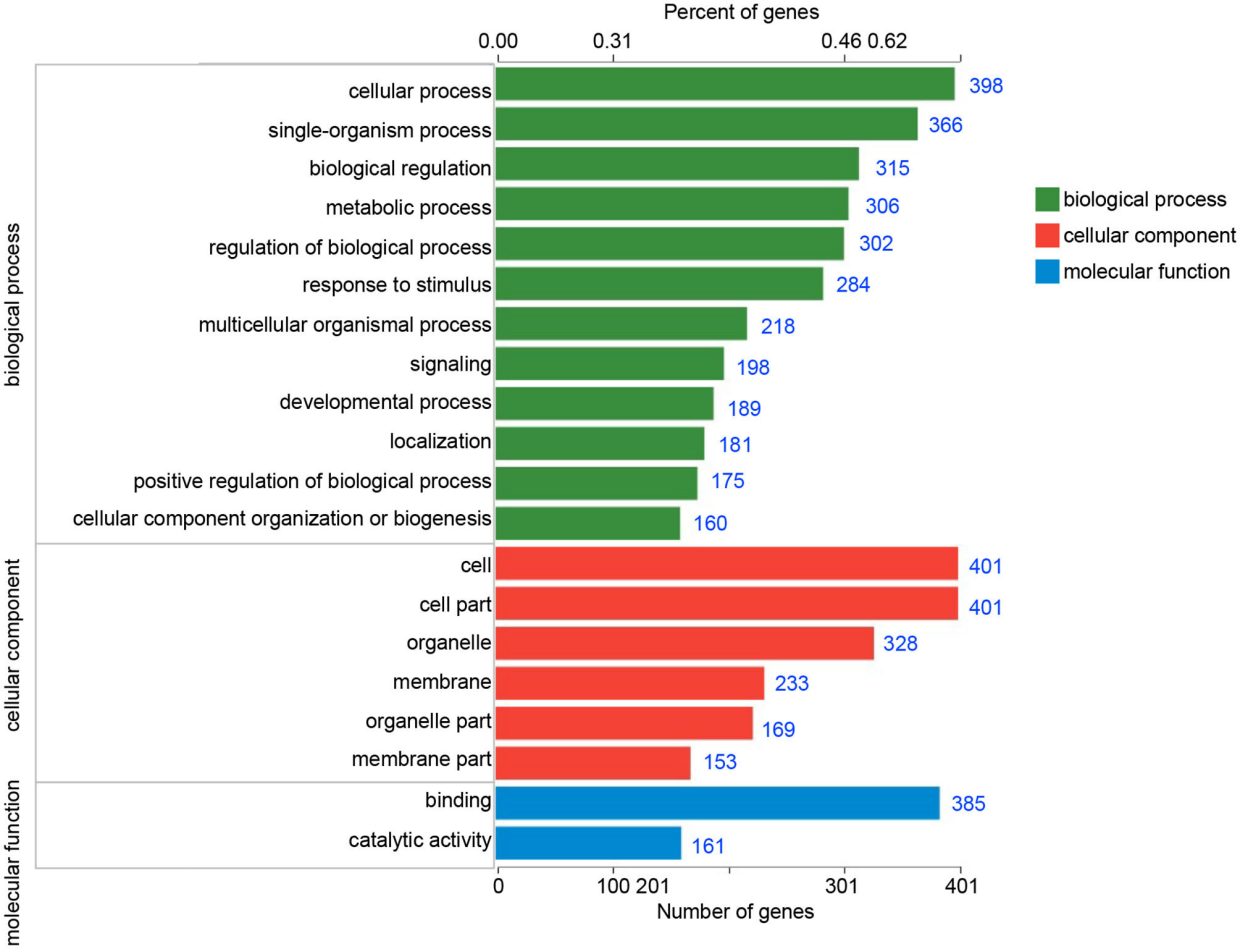
Gene ontology (GO) classifications of the differentially expressed genes (DEGs). All of the DEGs were assigned to three categories: biological process, cellular component, and molecular function. The blue number after each column represents the number of genes contained in the entry.

**Figure 8:**
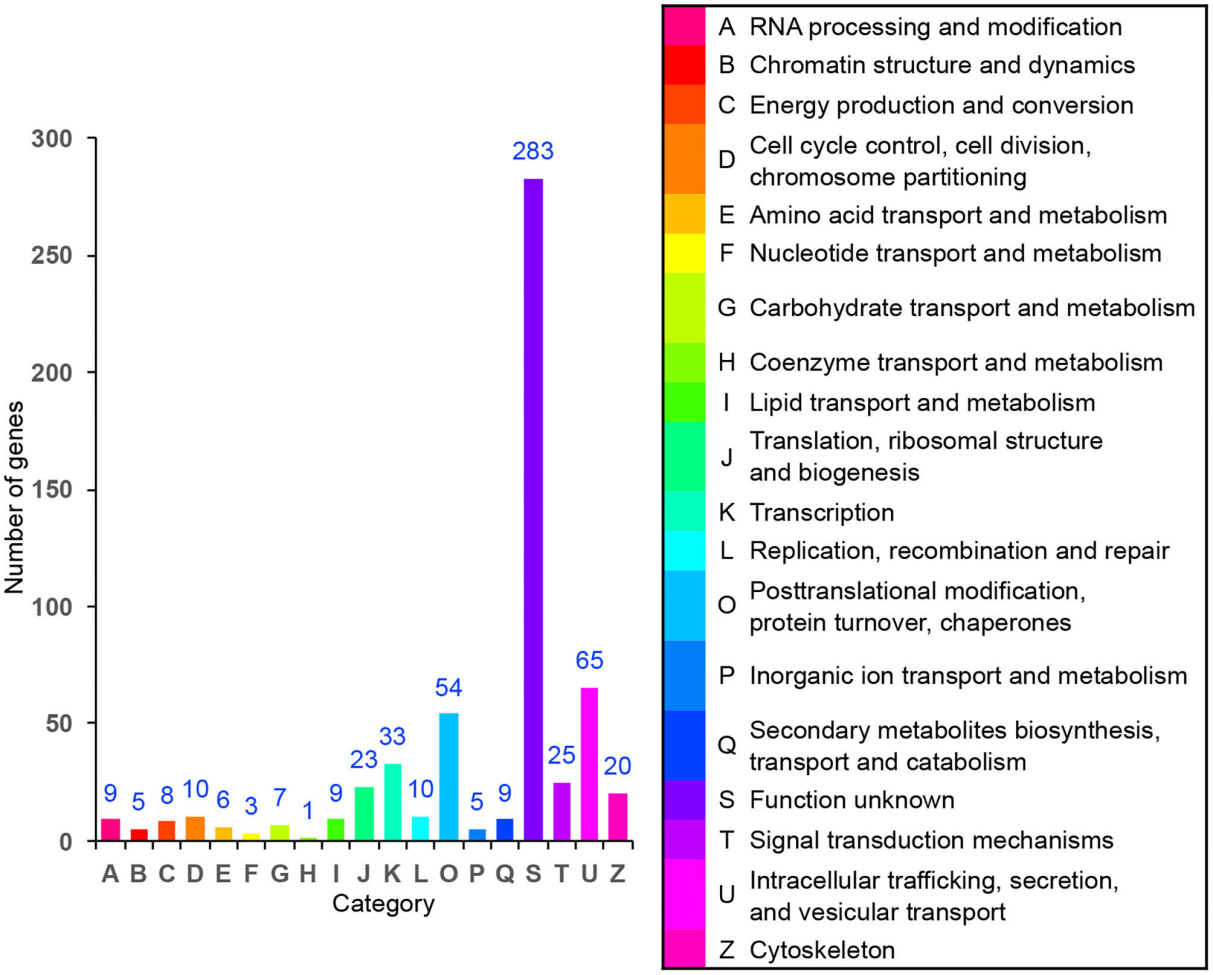
Cluster of orthologous group (COG) assignment of the differentially expressed genes (DEGs). The vertical axis represents the number of DEGs in each category, and the horizontal axis represents the COG functional category.

In order to identify the biological process by which LL-37 may regulate autophagy, HaCaT cells were transfected and treated with 1 μg of LL-37 plasmids or empty vectors for 24 h in triplicate. The total RNA of the HaCaT cells was extracted using the TRIzol UP reagent according to manufacturer's instructions. The Illumina HiSeq platform was used to undertake the high-throughput sequencing. As a result, we identified 650 DEGs between the cells transfected with LL-37 plasmids or empty vectors with fold changes of ≥2 or ≤0.5 and *P* < 0.05, of which 374 genes were upregulated and 276 genes were downregulated (Figure 6). GO assignments were used to assign a functional classification to these DEGs. All of the DEGs were annotated with 20 functional terms and categorized as a biological process, cellular component, or molecular function (Figure 7). COG was then used to assign a functional classification to these DEGs. After classification of the homology group database, U (Intracellular trafficking, secretion, and vesicular transport) was found to be the most representative functional cluster after S (Function unknown) (Figure 8).

### DEGs Involved in Autophagy

**Figure 9:**
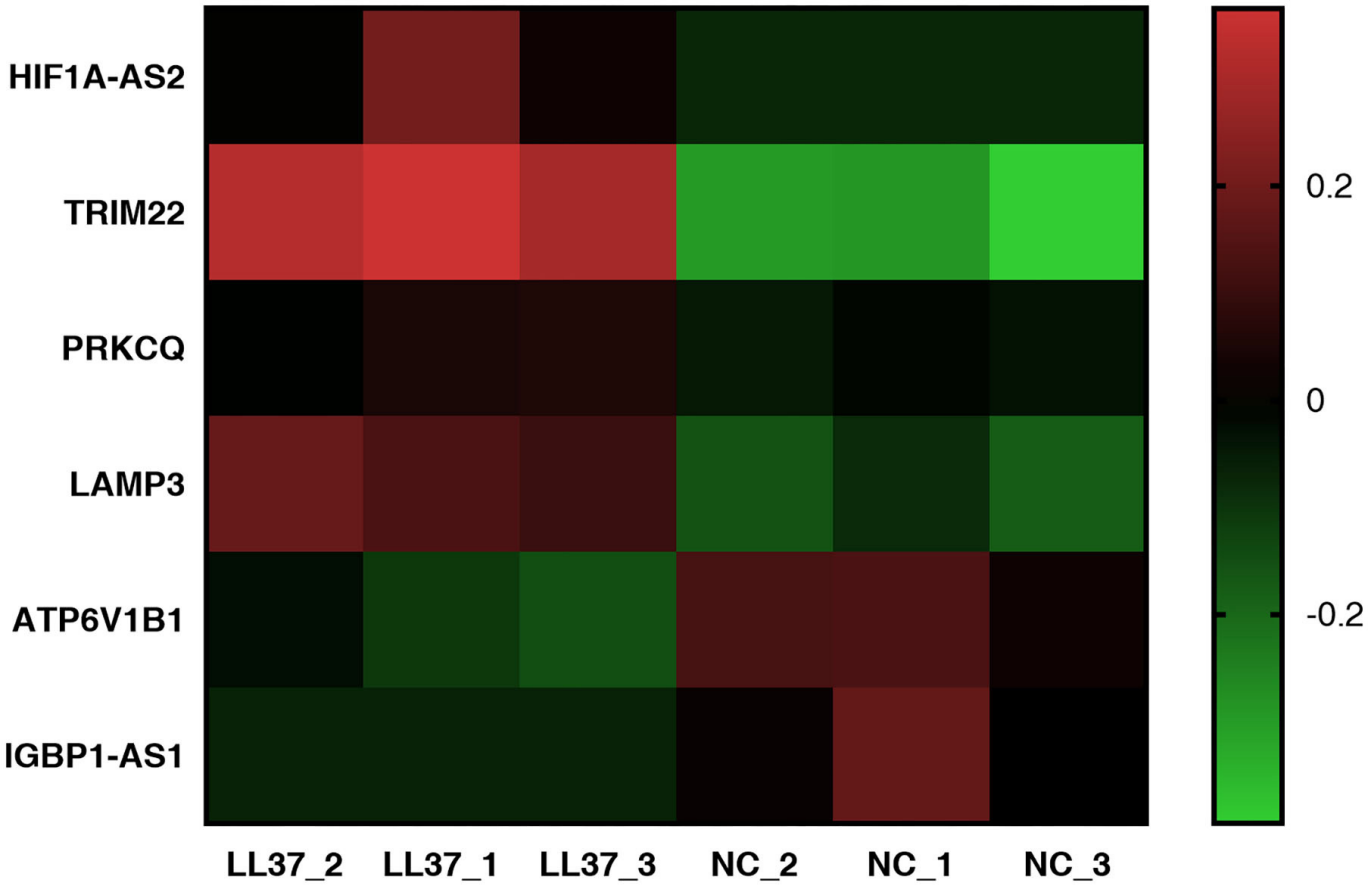
Heat map of autophagy-related differentially expressed genes (DEGs). The heat map was completed using GraphPad Prism 7. The vertical axis represents an autophagy-related gene, and the horizontal axis represents a sample. Up-expression is represented in red, and down-expression is represented in green.

We identified six candidate autophagy-related genes from all DEGs based on GO enrichment analysis and the KEGG database (Table 2, Figure 9). The qRT-PCR assays conveyed that the relative expression levels of the tripartite motif-containing 22 (*TRIM22*) and lysosomal-associated membrane protein 3 (*LAMP3*) mRNA in cells treated with LL-37 plasmids were 3.76 and 2.44 times that of the control, respectively, with a statistical significance *P* < 0.05.

**Table 2:** Details of autophagy-related differentially expressed genes (DEGs).

**Gene name**	**FCH (LL-37/NC)**	***P***	**Regulate**	**FCP (LL-37/NC)**
*HIF1A-AS2*	18.66	0.03	Up	1.40
*TRIM22*	5.31	0.00	Up	3.76
*PRKCQ*	2.94	0.01	Up	1.81
*LAMP3*	2.50	0.00	Up	2.44
*ATP6V1B1*	0.30	0.00	Down	0.71
*IGBP1-AS1*	0.06	0.03	Down	0.97

*HaCaT cells were transfected and treated with 1 μg of LL-37 plasmids (LL-37 group) or empty vectors (NC group) for 24 h in triplicate replicate. FCH (LL-37/NC): Fold changes of the DEGs between NC and LL-37 samples obtained by the high-throughput sequencing. FCP (LL-37/NC): Fold changes of the DEGs between NC and LL-37 samples detected by qRT-PCR*.

### PPI Network Formation

**Figure 10:**
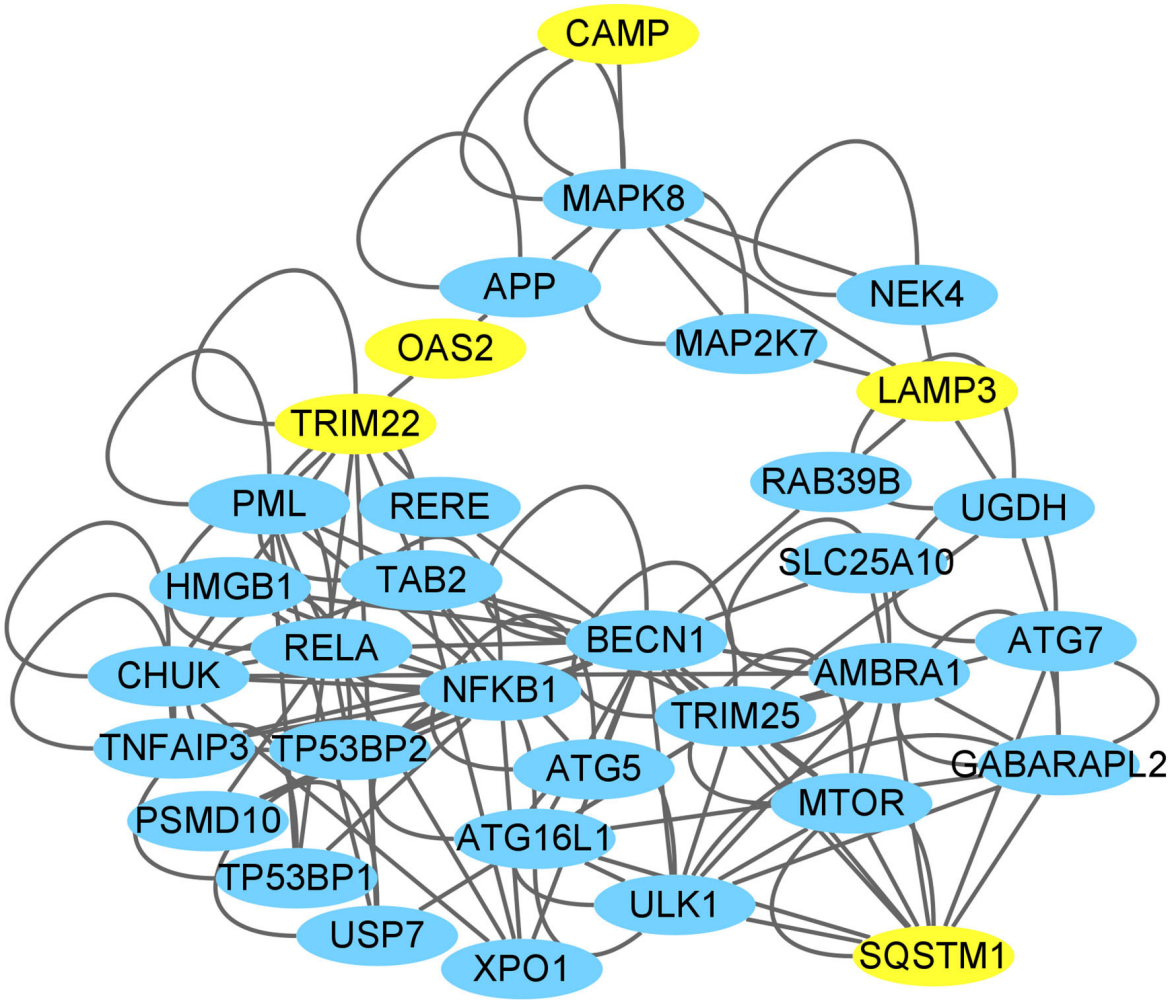
CAMP interaction with autophagy network suggested by the Cytoscape. The yellow nodes represent the up-regulated genes of the DEGs through high-throughput sequencing analysis or verification by the present experiments (SQSTM1/p62).

We investigated the connections between the CAMP and *TRIM22/LAMP3* gene and their protein product (also labeled TRIM22/LAMP3) as well as the networks of TRIM22/LAMP3 and their interaction with autophagy. The PPI network constructed by Cytoscape is shown in Figure 10. The network included 26 nodes and 80 edges, and the yellow nodes represent the upregulated genes of the DEGs.

## Discussion

Studies have found that *P. gingivalis* can internalize in a variety of host cells and is closely related to the occurrence and development of a variety of systemic diseases (Gibson et al., 2004; Kozarov et al., 2005; Karnoutsos et al., 2008; Zaitsu et al., 2016). Therefore, antagonizing *P. gingivalis* in host cells is helpful to control periodontitis and systemic diseases. In this study, we first found that LL-37 can reduce the number of live *P. gingivalis* internalized in keratinocyte HaCaT cells by promoting autophagy.

First, this study revealed that overexpression of LL-37 decreased the quantity of viable *P. gingivalis* internalized in HaCaT cells in a dose-dependent manner. LL-37 has functions such as anti-pathogenic microorganism activity and immunomodulatory activity (Lin et al., 2015; Svensson et al., 2016; Jiang et al., 2018). It also exhibits a broad spectrum of antibacterial activity against most Gram-positive and Gram-negative bacteria (Vandamme et al., 2012). However, studies have indicated that *P. gingivalis* has low sensitivity and even resistance to LL-37 (Altman et al., 2006), potentially as a result of the degradation of LL-37 by the virulence factor of gingipains and the low affinity of *P. gingivalis* to LL-37 (Bachrach et al., 2008). Researchers have also found that LL-37 could inhibit the number of *Mycobacterium tuberculosis* by promoting autophagy in macrophages (Rekha et al., 2015; Wan et al., 2018). Therefore, we speculated that LL-37 could induce autophagy to degrade *P. gingivalis* in the keratinocyte, which has not been proved or elucidated by prior studies.

The results showed that *P. gingivalis* significantly promoted autophagy in a concentration- and time-dependent manner in the HaCaT cells. However, the number of live *P. gingivalis* decreased noticeably after the inhibition of autophagy by 3-MA treatment. We therefore posit that autophagy may protect against the internalization of *P. gingivalis* in keratinocytes. Our previous study found that *P. gingivalis* could promote the formation of incomplete autophagosomes to protect them from elimination in non-phagocytes, such as epithelial cells, while *P. gingivalis* may induce functional autophagy in professional phagocytes, such as monocytes (Hu et al., 2019). Similar to the results above, the findings of our study indicate that *P. gingivalis* may survive in the keratinocytes by promoting imperfect autophagy.

The results of this study also suggested that LL-37 can promote autophagy process to eliminate *P. gingivalis* in cells. Our study found that LL-37 could significantly induce autophagy by increasing the ratio of LC3-II/LC3-I and decreasing the expression of p62 in HaCaT cells with concentration and time dependencies. Similar results were reported by Rekha et al. (2015) and Yuk et al. (2009). In addition, LL-37 induced autophagy in *P. gingivalis* internalized HaCaT cells. However, after autophagy was inhibited by 3-MA, the bacteriostatic effect of LL-37 on *P. gingivalis* decreased significantly, indicating that LL-37 reduces the number of live *P. gingivalis* in HaCaT cells by promoting autophagy. As we mentioned above, *P. gingivalis* has low sensitivity or even resistance to LL-37 as a result of the degradation of LL-37 by the virulence factor of gingipains or the low affinity of *P. gingivalis* to LL-37 (Ouhara et al., 2005; Altman et al., 2006; Bachrach et al., 2008; Gutner et al., 2009). Furthermore, Puklo et al. found a 11-kDa cathelicidin-derived fragment present in gingival crevicular fluid (GCF) that was collected from the pockets of patients with chronic periodontitis. This finding suggested that the bacterial proteases of *P. gingivalis* may degrade hCAP18/LL-37 to inhibit its antibacterial effect (Puklo et al., 2008). Therefore, we speculate that autophagy is a more important method by which LL-37 can inhibit the intercellular live *P. gingivalis* than direct bactericidal effects.

Furthermore, the transcriptome sequencing results indicated that LL-37 plays an important role in autophagy. The COG functional analysis was used to assign 65 DEGs to an “Intracellular trafficking, secretion, and vesicular transport” cluster. Vesicular transport is a cellular transport process by vesicle membranes. The inner membrane system refers to organelles surrounded by membrane structures including autophagosomes and lysosomes (Mellman and Warren, 2000; Bonifacino and Glick, 2004; Maxfield and van Meer, 2010). In addition, a GO analysis was used to assign DEGs into three main categories, in which “biological process” and “cellular component” were closely related to autophagy. Therefore, the COG functional analysis and GO analysis indicated the important roles of LL-37 in autophagy of HaCaT cells.

The transcriptome sequencing results and qRT-PCR assays showed that the gene *TRIM22* was significantly up-regulated in LL-37 treated cells. TRIM is involved in the regulation of almost all life activities (Tocchini and Ciosk, 2015). A large number of studies have found that TRIM can even regulate autophagy mechanism (Mandell et al., 2014; Kimura et al., 2015; Chauhan et al., 2016; Lou et al., 2018; Wang et al., 2018). As a member of the TRIM protein family, TRIM22 has been proved to induce autophagy (Kimura et al., 2015; Lou et al., 2018). It is worth noting that the findings of Lou et al. indicated that TRIM22 can regulate autophagy of THP-1 cells by up-regulating the NF-κB/Beclin-1 pathway, and eliminate intracellular *Mycobacterium tuberculosis* by promoting autophagy (Lou et al., 2018). Also, it has been shown that LL-37 can promote the expression of NF-κB (Suzuki et al., 2019) and the induction of transcription activity of NF-κB (Lim et al., 2015). In the HaCaT cells, we speculated that LL-37 may induce autophagy by TRIM22/NF-κB/Beclin-1 pathway, which remains to be proved in future experiments.

In addition to *TRIM22*, LL-37 significantly up-regulated the expression of gene *LAMP3*. LAMP3 is the third member of the LAMP family (de Saint-Vis et al., 1998) and plays a vital role in autophagy (Tanaka et al., 2000; Eskelinen et al., 2002). The LAMP protein is thought to be involved in the fusion of autophagosomes and lysosomes into autophagolysosomes (Tanaka et al., 2000; Zheng et al., 2011). Nagelkerke et al. indicated that LAMP3 is localized to the lysosomal membrane and is involved in the fusion of autophagosomes and lysosomes in breast cancer cells. After LAMP3 knockout, autophagy was suppressed (Nagelkerke et al., 2014). In a future study, we will try to explore the potential LAMP3-associated pathway through which LL-37 induces autophagy in keratinocytes.

The PPI network showed that CAMP could interact with the proteins translated from the up-regulated genes *TRIM22/LAMP3* through MAPK8. It is worth noting that the KEGG enrichment analysis revealed the MAPK signaling pathway as one of the most significantly enriched pathways in our study. Furthermore, in this network, some molecules have been reported to be regulated by TRIM22 and LAMP3 to promote autophagy, such as NF-κB, Beclin-1, and Atg 5 (Wang et al., 2011; Takaesu et al., 2012; Qiu et al., 2013; Seto et al., 2013; Huttlin et al., 2017). However, most molecules in the predicted network are not the DEGs identified in our study. The other possible reason may be that the related molecules could promote autophagy by their enhanced functions (such as an increase in phosphorylation efficiency) rather than by expression level changes.

The limitation of this experiment was that LL-37 knockdown was not performed on HaCaT cells. The main reason was the low expression of endogenous LL-37 in HaCaT cells, which has been supported by other studies (Svensson et al., 2016; Jiang et al., 2018; López-González et al., 2018; Suhng et al., 2018). In addition, cells that were transfected with LL-37 plasmids could not suppress the expression by knockdown. At present, there are no effective antagonists or neutralizing antibodies of LL-37 to antagonize the effect of LL-37. Therefore, cell lines with a high expression of endogenous LL-37 should be screened in a future study. In addition, the related molecular pathway of LL-37-induced autophagy during the *P. gingivalis* elimination process should be explored further.

In conclusion, our findings indicated that LL-37 can reduce the number of live *P. gingivalis* internalized in keratinocytes by promoting autophagy. The prediction of transcriptome sequencing and the verification assay suggested that LL-37 plays an important role in autophagy and might promote autophagy of keratinocytes by regulating *TRIM22* and *LAMP3*. This study provides scientific clues about the role and potential application of LL-37 in the elimination of *P. gingivalis* in keratinocytes, and in turn, it can be used to develop methods for the prevention of periodontitis treatment of associated diseases.

## Data Availability Statement

The RNA-seq data has been submitted to SRA database in NCBI, BioProject number PRJNA663720, accessions SRR12649922 - SRR12649927.

## Author Contributions

XT, YP, XF, JL, YG, and XY designed the study. XY performed the experiments with the help from XT. JL, YG, LN, FG, and XY wrote the final manuscript. XT, YP, XF, LN, CP, and YG revised the manuscript. All authors contributed to the article and approved the submitted version.

## Conflict of Interest

The authors declare that the research was conducted in the absence of any commercial or financial relationships that could be construed as a potential conflict of interest.

## Abbreviations

*P. gingivalis*: *Porphyromonas gingivalis*
hCAP18: human cationic antimicrobial peptide-18
NF-κB: nuclear factor-kappa B
MAPK: mitogen-activated protein kinase
BHI: brain heart infusion
CFU: colony forming unit
MOI: multiplicity of infection
qRT-PCR: quantitative real time-polymerase chain reaction
GO: gene ontology
KEGG: kyoto encyclopedia of genes and genomes
COG: cluster of orthologous group
TRIM22: tripartite motif-containing 22
LAMP3: lysosomal-associated membrane protein 3.
